# Awareness of and interaction with physician rating websites: A cross-sectional study in Austria

**DOI:** 10.1371/journal.pone.0278510

**Published:** 2022-12-30

**Authors:** Bernhard Guetz, Sonja Bidmon

**Affiliations:** Department of Marketing and International Management, Alpen-Adria-Universitaet Klagenfurt, Klagenfurt am Wörthersee, Austria; Duervation, AUSTRIA

## Abstract

To date, the digital assessment of service experiences represents a decisive process step of a feedback culture in numerous economic areas. In view of this digitalization of service assessments, the importance of Physician Rating Websites (PRWs) has also increased steadily in recent years. Even though these websites could be perceived as a powerful communication tool for the exchange of health specific information, the knowledge about whether and how different population segments use these portals has been limited so far. For this reason, our aim was to investigate the level of awareness regarding PRWs among the study population and to discover how users interact with this specific type of online portals. We performed an online survey including 558 participants. To ensure the attention and integrity of participants, attention checks were included in the questionnaire. Study participants who did not exceed the mentioned security levels were excluded from the study. Statistical analyses were carried out, using IBM SPSS Statistics 27. To illustrate the relationship between demographic variables and dependent variables, two tailed chi square tests were performed. Comparison of means and t-testing was used to investigate the relationship between psychographic variables and the dependent variables. In addition to that, the awareness levels regarding different rating portals were evaluated using descriptive methods. Our results suggest that the general awareness regarding PRWs is relatively high (75.6%, 423/558), especially among female (x^2^_1_ = 9.880, P = .002), middle-aged (x^2^_9_ = 26.810, P = .002), more highly educated (x^2^_4_ = 19.038, P = .001), urban (x^2^_1_ = 6.274, P = .012), digitally literate (t_203_ = 2.63, P = .009) individuals and particularly among respondents with a higher eHealth literacy (t_203_ = 2.37, P = .019). Even though more than three quarters of the respondents know that PRWs exist, compared to other rating platforms, they are only in the lower midfield. The upper ranks are taken by websites on which restaurant visits (98.9%, 552/558), hotel stays (97.7%, 545/558) or movies (95.5%, 533/558) can be rated. The most popular PRWs in Austria include Docfinder.at (31.3%, 175/558; 77.8%, 434/558) followed by the evaluation tools provided by Google.at (8.24%, 46/558; 70.3%, 392/558) and Herold.at (1.61%, 9/558; 44.8%, 250/558). In Austria, PRWs seem to be characterized by a high degree of interaction (89.2%, 498/558) with a wide variety of different types of interactions. While many respondents use PRWs to retrieve general information (83.2%, 464/558), there are significantly fewer who read physicians’ reviews (60.9%, 340/558) and use this portal to select a physician (60.6%, 338/558). Respondents who have already rated a doctor themselves belong to the smallest group accounting for just 14.7% (82/558). Significant effects regarding the interaction with PRWs exist between different genders, ages, education levels, marital statuses, occupations and areas of living. In addition to that, respondents with better feelings towards the internet, greater digital literacy as well as a higher eHealth literacy were also characterized with a higher interaction rate regarding PRWs. According to the high level of awareness of and interaction with PRWs within our study population, PRWs appear to be a successful medium for health-related communication. Especially for female, middle-aged, more highly educated, urban and more technology savvy population segments, PRWs seem to represent an effective tool to support the health-specific decision-making process.

## Introduction

Due to the increasingly competitive environment in the private and public health sector, the importance of customer orientation in health management has grown steadily in recent years [1]. In addition to that, the rise in patient orientation has also led to patients being more involved in the medical decision-making process [2]. These relative factors are a clear signal of the increasing importance of patients as opinion leaders [3–5]. Traditionally, the role as opinion leader has been particularly important within the family association and circle of friends [6–9]. Nevertheless, with the rise of the internet and network technology, this rather limited circle of stakeholders has expanded considerably [10–12]. The opportunities offered by these technologies enable people to network and exchange information detached from time and residence or location [13–15]. Moreover, this modern type of information exchange does not stop when it comes to information regarding the healthcare sector [16–18].

In this context, Physician Rating Websites (PRWs) could be a promising means for health-specific communication purposes [19]. These online portals allow users to discuss, rate and comment on physicians’ medical and service quality in a social media setting [20]. In this context, PRWs offer users the possibility to generally inform themselves about the expected medical service and allow them to choose between different alternatives according to their personal needs [21]. Additionally, the importance of PRWs has grown steadily in recent years [22], albeit at a much lower rate than was expected [23]. In analogy to the increasing societal attentiveness towards PRWs, the related scientific interest has also evolved steadily during the recent past [22–38]. Amongst others, this fact is represented in the growing number of different studies from regions all over the world like North America [20, 24, 39–52], Europe [1, 33, 36, 51, 53–65], Asia [66–74] or Australia [75–77]. Within such investigations, a wide range of research areas was examined, such as the frequency of ratings [36, 77], the effects of negative reviews [20, 48, 70], the content of reviews [1, 24, 39–47, 49, 53, 57, 63, 66–69, 71–76], factors that have an impact on the evaluation behavior [56, 61, 64], or the behavior of physicians [62]. Another investigated area in this context is patients’ awareness of and interaction with PRWs. In this context, different studies from England [54, 65], the United States [50, 52, 78], Germany [33, 51, 58] and Switzerland [28] could be identified. Through literature search, we were even able to identify studies by Austrian research institutions on awareness of and interaction with PRWs, but these studies were conducted with a German data set [1, 56, 58]. Although the same language is spoken in Austria and Germany, the health care systems differ substantially and it cannot be assumed that research results are comparable between Austria and Germany with regard to the awareness and use of PRWs. This conclusion is based on cultural differences with regard to various aspects like corporate governance [79–81], multiculturalism [82], but also with regard to health-specific perception [83] and the integration of online media and online tools in health care [84, 85].

Within the studies already performed, different factors that seemed to be related to health-specific technology use, like the usage of PRWs, could be identified. These include demographic variables such as gender, age, education, marital status and occupation [33, 51, 54, 58]. Furthermore, different psychographic criteria that seem to be related to the use of health-specific digital technologies were discovered. Among them are eHealth literacy, digital literacy as well as the feelings towards the internet [30, 58, 86]. In this context, eHealth literacy refers to a person’s capacity to look for, comprehend, and evaluate health information from online resources and to apply that knowledge to an issue regarding their personal health [87]. Digital literacy, by comparison, is the ability to use digital technologies to solve a variety of different challenges in daily life [88]. In contrast to digital literacy, eHealth literacy focuses exclusively on the use of digital technologies to search for and use information in a health-specific environment. Finally, feelings towards the internet describe the general attitude of people towards the internet as well as towards web-based applications [58].

Moreover, there seem to be at least two further factors that potentially affect the process of engaging with these portals. PRWs can be seen as a specific form of electronic Word-of-Mouth [89] and therefore represent a kind of substitute for recommendations, which are usually given within personal interactions. This substitution might be more important in regions characterized by a higher degree of resident anonymity like urban regions [90] as compared to rural ones, which might lead to a difference between rural and urban regions with regard to the prevalence of usage of PRWs. In addition to that, the level of skepticism towards online reviews (*review skepticism*) could also negatively influence the engagement with PRWs [29].

The above-mentioned facts show that there has been a research gap with regard to the awareness of and interaction with physician rating websites in Austria so far. For this reason, this paper addresses four distinct research questions with regard to awareness of and interaction with PRWs in Austria. They are elaborated in detail in the next two sections.

### Awareness regarding physician rating websites (RQ1 and RQ2) and brand awareness of specific PRWs (RQ3)

*Awareness* can occur in different manifestations. In common parlance, *awareness* is defined as mental perception of the existence of a product, a service, a brand or a particular situation [91]. A similar conceptualization of *awareness* can also be found in a number of related publications that examine the *general awareness of PRWs* [33, 51, 54, 58]. For this reason, we investigate how the *awareness regarding PRWs* is distributed according to different sociodemographic and psychographic segments within the study population (RQ1).

In addition to that, the awareness regarding PRWs can further be compared to the awareness gained by rating portals in other realms of daily life like hotel rating portals or employer rating portals. One published study chose this procedure and compared the *awareness regarding PRWs* with the *awareness regarding alternative online rating portals* [50]. Accordingly, RQ2 investigates the level of *awareness regarding PRWs* contrasted to *alternative online rating portals*.

Another form of awareness, frequently examined in advertising studies, is *brand awareness*. For consumers, *brand awareness* plays a crucial role within their individual decision-making process [92]. In this context, a distinction can consistently be found between the two well-known types of brand awareness, i.e. *brand recall (recall)* and *brand recognition (recognition)* [93]. *Recall* refers to the retrieval of a brand name or symbol that has been stored in an individual’s memory in the past [94]. In contrast to that, *recognition* describes the ability to remember things previously experienced when they are displayed or mentioned [95]. In this context, PRWs can also be understood as brands, as they often feature their own brand name and brand logo. The focus is thus on the extent to which individuals can recall and recognize the names and respective brands of distinct Austrian PRWs. Therefore, the third purpose of our study is to investigate the *brand awareness* (*recall* and *recognition*) of distinct Austrian PRWs according to the study population (RQ3).

### Interaction with physician rating websites (RQ4)

*Interaction* is understood as an occurrence in which individuals or objects convey or respond to each other [91]. If computers are involved, the term *human computer interaction* (HCI) is used to refer to the actions that a user performs on a computer [96]. A particular form of HCI is the human website interaction, which describes how individuals interact with certain websites [97]. In the area of PRWs, there are different possibilities of interaction, which are variously expressed depending on the functionality of the respective website. In general, PRWs commonly contain information regarding physicians’ practices like opening hours, telephone number or the address, which shows that those websites could serve as a source of *general information* [19]. When it comes to the evaluation aspect, however, users can generally choose between *reading patient ratings* of physicians and *providing personal feedback* on physicians [20]. From a decision supporting perspective, PRWs probably serve only two purposes from the patients’ perspectives, which are the search for physicians and the selection of future physicians [35, 41, 98]. In this context, our fourth research question is:

RQ4: How do patients interact with PRWs and what are the driving factors for distinct usage of PRWs?

## Methods

The present study did not require approval of an ethics board in Austria, according to section 8c of the Federal Hospitals and Health Care Institutions Act as well as section 30 of the Universities Act. Corresponding to this legal framework, consultation of an ethics board is obligatory only for clinical trials of medicinal products and medical devices, as well as for the application of new medical methods and applied medical research on humans. Since the study scope is not health related, the study design and data collection did not require ethical approval. No sensitive issues were raised within the questionnaire and the evaluation method did not allow any conclusions to be drawn about the person who had responded to the survey. In addition, the questions were kept very general and there was at no time a risk of harm in any form by answering them. No sensitive data were requested and all panel members gave their consent to the data collection. The study was conducted by usage of the crowdsourcing platform Clickworker.com. All projects and orders are subjected to an auditing process before being accepted. In this procedure, trained employees check whether the survey is feasible, whether personal data is requested and whether discriminatory or unethical content occurs. Orders in which personal data are requested, or include discriminatory or unethical content are not accepted. Participants were informed and appropriately instructed about their voluntary involvement in an online survey, as well as the fact that their data would be treated with absolute confidentiality using suitable methods, procedures, and protocols. Individuals who agreed to participate voluntarily were informed about the survey in writing before and after completing the questionnaire. The data was processed in a strictly confidential and anonymous manner [99].

### Questionnaire design and mode

The questionnaire used for the current study was designed by using existing and validated scales. The sociodemographic variables were taken from Bidmon et al. [15] and the respective items were used to measure gender, age, education level, and marital status. Occupation was measured using a 9 item scale adapted from Emmert et al. [51]. Within the area of living part, the survey participants were asked whether they lived in a more urban or a more rural area. In this case, the two item scale of Liff et al. [100] was used as a template. Feelings towards the internet and digital literacy were both measured on a seven-point scale taken from Terlutter et al. [58]. In addition to that, eHealth literacy was measured using the German version of the seven item eHealth literacy scale developed by Norman & Skinner [101], which has been translated into German and published by Soellner et al. [102]. Finally, the psychographic variable review skepticism was measured on a four item scale developed by Skarmeas & Leonidou [103]. In the second part of the questionnaire, general information regarding the participants’ knowledge about online rating and review websites was gathered. To investigate the awareness of PRWs in comparison with alternative rating portals, the multiple response scale adapted from Hanauer et al. [50] was used. In addition to that, within this questionnaire area, Keller’s Brand Recall scale [104] was adapted for the study focus of PRWs. The third questionnaire section commenced with the attention check. Participants had to select a specific answer, defined in the introductory text, in order to proceed with the questionnaire. The attention check was created following Oppenheimer et al. [105] and Kung et al. [106]. The attention check was followed by the question regarding brand recognition. This multiple response scale that asked for knowledge regarding different PRW brands was taken from Emmert et al. [51] and adapted to the Austrian market. Finally, the degree of interaction with PRWs was assessed using a multiple response scale developed by Burkle & Keegan [20]. The original scale was extended to include the categories search for physician(s), select physician(s), and search for additional information. The questionnaire and the justification of the items can be found in the supporting information section (S1 File).

All items used were translated/back translated by both, an English and a German native speaker who each had fluent language skills in the foreign language. In order to identify potential ambiguity or uncertainty, a pre-study with 20 participants was performed. After slight modifications as well as the implementation of an ‘other’ option within some items, the adapted version of the questionnaire was used to carry out the main study.

### Data collection procedure

As mentioned above, the data collection was performed by using a specific online survey tool (Clickworker.com) which is a crowdsourcing platform for research similar to Amazon’s Mechanical Turk (MTurk), however even more widespread in Europe. Clickworkers voluntarily participate in the available surveys, whereby they only see those surveys for which they are approved via a profile filter. This filter is defined by the entries in the order (age, gender, country, native language,….). All clickworkers from Austria between the ages of eighteen and ninety-nine were able to take part in the survey. Participation was possible via the desktop application as well as via the Android and iOS app. Participants were recruited by making the survey accessible within the system for all persons who met the criteria mentioned above. We followed recommendations to mitigate validity threats when using crowdsourcing platforms (see e.g., [107]) by including 3 security levels in the online questionnaire. First, the attention and integrity of survey participants was ensured by logic tasks and attention checks. Before participants were able to start answering the questions, they had to solve a mathematical equation to prove that they are human as well as confirming their eligibility. Furthermore, an attention check was included. Within this attention check, participants had to select a certain answer option within a specific question to prove that they had read the introduction text of the third question group [105, 106]. Finally, a cookie was set for each participant to prevent repeated participation. In addition to these security levels, the order of possible answers varied for knowledge related questions in each questionnaire cycle. An example equation as well as the applied introduction text and the description of the attention check can be found in the supporting information section (S2 File).

### Data preparation

The survey data was obtained by using the survey tool Lime Survey. Since the settings of the questionnaire instrument did not allow for non-answered questions in the area of awareness and interaction with PRWs, there was no missing data within the survey.

### Data analysis

Statistical analyses were conducted by using IBM SPSS Statistics 27. Two-tailed chi-square tests were used to illustrate the correlation between demographic characteristics (e.g. age or gender) and dependent variables (e.g. general awareness or type of interaction). Moreover, we used comparisons of means and t-testing to show correlations between psychographic characteristics (e.g. feelings towards the internet or eHealth literacy) and the key variables (e.g. general awareness or type of interaction). Finally, brand recall and recognition were contrasted by descriptive methods.

## Results

The initial survey sample consisted of 852 respondents. As a first data cleansing step, the exclusion of those participants who had not passed the attention check led to a reduction of the total sample to n = 613. Another thorough data check controlling for extremely short answer times, flatliners and inconsistent answer patterns led to a final survey sample of n = 558 respondents. The described data selection process is illustrated in the supporting information section (S3 File). A sample description can be found in Table 1.

**Table 1:** Sample description with regard to sociodemographic variables (n = 558).

*Sociodemographic characteristics*	*Absolute Frequencies (Number of respondents) (n)*	*Relative Frequencies (Percentage of respondents) (%)*
***Gender***		
*Female*	306	54.8
*Male*	250	44.8
*Diverse*	2	.4
***Age***		
*15–19*	75	13.4
*20–24*	152	27.2
*25–29*	106	19.0
*30–34*	78	14.0
*35–39*	64	11.5
*40–44*	34	6.1
*45–49*	16	2.9
*50–54*	16	2.9
*55–59*	9	1.6
*60+*	8	1.4
***Education***		
*Compulsory education*	32	5.7
*Vocational secondary education*	62	11.1
*Apprenticeship*	76	13.6
*High school*	232	41.6
*University degree*	156	28.0
***Marital Status*** *		
*Single*	226	40.5
*Close-partnered*	229	41.0
*Married*	93	16.7
*Divorced*	10	1.8
***Occupation***		
*Salaried employee*	256	45.9
*Unemployed*	49	8.8
*Self-employed*	52	9.3
*In training (pupil/student)*	193	34.6
*Retired*	8	1.4
***Area of Living***		
*Urban*	346	62.0
*Rural*	212	38.0

*There were no widowed participants within our study sample

### Results on awareness (RQ1–RQ3)

The following section summarizes our results on awareness regarding PRWs. In this context, a distinction is made between the three following forms of awareness: *general awareness* (RQ1), *awareness compared to other rating portals* (RQ2) and *brand awareness* (RQ3).

RQ1: In total 75.6% (422/558) of our study participants were aware that PRWs exist. Statistically significant effects regarding the *general awareness* of PRWs were found within the demographic characteristics of gender (x^2^_1_ = 9.880, *P* = .002, V = .13), age (x^2^_9_ = 26.810, *P* = .002, V = .22), education (x^2^_4_ = 19.038, *P* = .001, V = .19), occupation (x^2^_4_ = 10.937, *P* = .027, V = .14) and area of living (x^2^_1_ = 6.274, *P* = .012, V = .11). Table 2 summarizes the characteristics of the individual relationships between the variables.

**Table 2:** Coherence of demographic variables and general awareness regarding PRWs.

*Variables*	*Aware n (%)*	*Not Aware n (%)*	*Total*	*X ^2^ (df)*	*P*	*V*
***Gender*** ^a^				**9.880 (1)**	**.002**	**.13**
*Female*	247 (80.7)	59 (19.3)	306			
*Male*	173 (69.2)	77 (30.8)	250			
***Age***				**26.810 (9)**	**.002**	**.22**
*15–19*	46 (61.3)	29 (38.7)	75			
*20–24*	113 (74.3)	39 (25.7)	152			
*25–29*	78 (73.6)	28 (26.7)	106			
*30–34*	65 (83.3)	13 (16.7)	78			
*35–39*	60 (93.8)	4 (6.2)	64			
*40–44*	25 (73.5)	9 (26.5)	34			
*45–49*	12 (75.0)	4 (25.0)	16			
*50–54*	11 (68.8)	5 (31.2)	16			
*55–60*	8 (88.9)	1 (11.1)	9			
*60+*	4 (50)	4 (50)	8			
***Education***				**19.038 (4)**	**.001**	**.19**
*Compulsory Ed.*	18 (56.3)	14 (43.7)	32			
*Voc. Sec. Ed.*	46 (74.2)	16 (25.8)	62			
*Apprenticeship*	60 (78.9)	16 (21.1)	76			
*High School*	164 (70.7)	68 (29.3)	232			
*Univ. Degree*	134 (85.9)	22 (14.1)	156			
***Marital Status***				**.616 (3)**	**.893**	**.03**
*Single*	167 (74.3)	58 (25.7)	225			
*Close-partnered*	175 (76.4)	54 (23.6)	229			
*Married*	72 (77.4)	21 (22.6)	93			
*Divorced*	7 (70.0)	3 (30.0)	10			
***Occupation***				**10.937 (4)**	**.027**	**.14**
*Salaried employee*	210 (82.0)	46 (18.0)	256			
*Unemployed*	34 (69.4)	15 (30.6)	49			
*Self-employed*	38 (73.1)	14 (26.9)	52			
*In Training*	134 (69.4)	59 (30.6)	193			
*Retired*	6 (75.0)	2 (25.0)	8			
***Area of Living***				**6.274 (1)**	**.012**	**.11**
*Urban*	274 (79.2)	72 (20.8)	346			
*Rural*	148 (69.8)	64 (30.2)	212			

^a^ = without diverse gender (n = 2)

Significant effects were found for the investigated psychographic variables *digital literacy* and *general awareness* (t_203_ = 2.63, p = .009) as well as between *eHealth literacy* and *general awareness* (t_203_ = 2.37, p = .019). Table 3 shows the differences within the mean values of the psychographic variables and the results of significance testing (t-tests).

**Table 3:** Coherence of psychographic variables and general awareness regarding PRWs.

*Variables*	*Aware (n = 422)*		*Not Aware (n = 136)*		*T (df)*	*P*
	Mean	SD	Mean	SD		
Feelings toward the internet*	5.48	1.16	5.32	1.11	1.35 (556)	.177
Digital literacy**	6.24	.91	5.97	1.06	2.63 (203)	.009
eHealth literacy**	5.50	.97	5.24	1.13	2.37 (203)	.019
Review skepticism***	4.07	1.27	4.20	1.38	-1.04 (556)	.300

* The scale options range from 1 (“very negative feelings”) to 7 (“very positive feelings”).

** The scale options range from 1 (“not literate at all”) to 7 (“very literate”).

*** The scale options range from 1 (“not sceptic at all”) to 7 (“very sceptic”).

RQ2: In order to show differences within popularity levels regarding different online rating websites, we related the *general awareness* regarding PRWs to the awareness pertaining to alternative rating portals. Within our sample, the most pronounced online rating option was the rating of restaurants. 552 out of 558 (98.9%) study respondents indicated that they are aware of the possibility to rate their experiences with specific restaurants online. This was subsequently followed by the possibility to evaluate experiences regarding hotels 97.7% (545/558), movies 95.5% (533/558), and books 91.0% (508/558). As shown above, 75.6% (422/558) of the study participants were aware that PRWs provide the possibility to evaluate physicians online and a further 248 out of 558 (44.6%) respondents were aware that hospital rating websites exist. Table 4 represents the individual items including the respective awareness scores.

**Table 4:** Awareness of rating websites.

Are you aware that there are websites that rate and review the following?	Yes		No	
	Frequencies (n)	Percentages (%)	Frequencies (n)	Percentages (%)
Restaurants	552	98.9	6	1.1
Hotels	545	97.7	13	2.3
Movies	533	95.5	25	4.5
Books	508	91.0	50	9.0
Electronics assembly	496	88.9	62	11.1
Apparel	431	77.2	127	22.8
Leisure Activities	422	75.6	136	24.4
Physicians	422	75.6	136	24.4
Automotive	414	74.2	144	25.8
Employers	410	73.5	148	26.5
Real Estate	341	61.1	217	38.9
Teachers	272	48.7	286	51.3
Hospitals	249	44.6	309	55.4

n = 558

RQ3: Within the third awareness analysis, we try to describe the market penetration in terms of *brand awareness* for individual PRWs. *Recall* was the highest for Docfinder.at (31.36%) followed by the review pages of Google.at (8.24%) and Herold.at (1.61%). In addition to the valid responses regarding PRWs for the Austrian market, a number of non-valid websites were mentioned. These include portals that do not have a rating function for medical services (e.g., Tripadvisor.at) as well as websites on which it is not possible to rate or review Austrian physicians (e.g., Jameda.de). An overview of the recall rates for the individual responses can be found in Table 5.

**Table 5:** PRW brand recall.

Physician Rating Website	Recall Frequencies (n)	Recall Percentages (%)
Docfinder.at	175	31.36
Google.at (as PRWs)	46	8.24
Herold.at (as PRWs)	9	1.61
Gutgemacht.at	35	.90
Arztsuche24.at	3	.54
Praxisplan.at	2	.36
Doctena.at	1	.18
n = 558		

Other websites mentioned

(websites that are not available for the evaluation of physicians)

www.arzt.at, www.aerzteverzeichnis.at, www.arztnavigator.at, https://meddoctor.com.br/, https://online.medunigraz.at/, https://my-doc.com/, http://onlineapotheke.at/, https://www.tripadvisor.at/, http://www.webmed.at/, https://www.doccheck.com/, https://www.netdoktor.at/

Other websites mentioned

(websites that are not available for the evaluation of Austrian physicians)

https://www.aerzteblatt.de/, https://www.arzt-auskunft.de/, https://www.meinaerzteportal.de/aerzteportal.html, https://www.doccheck.com/, https://www.docinsider.de/, https://www.doctolib.de/, https://www.aerzte.de/, https://www.jameda.de/, https://www.sanego.de/

A similar pattern could be demonstrated for *recognition*. Most respondents recognized Docfinder.at (78.10%) as well as the review sections of Google.at (68.40%) and Herold.at (44.8%) as potential PRWs. Detailed information regarding the brand recognition survey part can be found in Table 6.

**Table 6:** PRW brand recognition.

Physician Rating Website	Recognized		Not Recognized	
	n	%	n	%
Docfinder.at	434	77.8	124	22.2
Google.at (as PRWs)	392	70.3	166	29.7
Herold.at (as PRWs)	250	44.8	308	55.2
FirmenABC.at (as PRWs)	175	31.4	383	68.6
Trustpilot.com	104	18.6	454	81.4
Arztsuche24.at	100	17.9	458	82.1
Doc-suche.at	92	16.5	466	83.5
Praxisplan.at	50	9.0	508	91.0
Yelp (as PRWs)	45	8.1	513	91.9
Gutgemacht.at	26	4.7	532	95.3
Tupalo.at (as PRWs)	22	3.9	536	96.1
Susi.at (as PRWs)	15	2.7	546	97.3
Doctena.at	8	1.4	550	98.6
Die-arztempfehlung.com	6	1.1	552	98.9
Mooci.org	2	.4	556	99.6

n = 558

### Results on interaction (RQ4)

RQ4: Most study participants 498/558 indicated that they had interacted with PRWs at least once in the past, i.e., 89.2% of our study respondents can be classified as users of PRWs. However, there were considerable differences in the types of interaction in which respondents engaged with regard to PRWs. In this context, 83.2% (464/558) of participants indicated that they had used PRWs to *search for general information* such as the address or the telephone number of a specific physician. In addition to that, 76.5% (427/558) of respondents used those websites to *search for physicians* followed by 60.9% (340/558) respondents who indicated that they use those websites to *read physicians’ ratings*. 60.6% (338/558) of respondents indicated that they use PRWs to *select future physicians*. Finally, 14.7% (82/558) of respondents reported that they had *provide*d *personal feedback* by rating a specific physician on PRWs at least once in the past.

Significant effects regarding the search for general information exist between females and males (x^2^_1_ = 4.902, *P* = .027) and different age categories (x^2^_9_ = 17.483, *P* < .001). Regarding the utilization of PRWs to *search for physicians*, significant effects were demonstrated for gender (x^2^_1_ = 6.922, *P* = .009), age (x^2^_1_ = 33.638, *P* = < .001) and occupation (x^2^_4_ = 10.723, *P* = .030). Moreover, regarding the use of PRWs to *read physicians’ ratings*, significant effects were found between different genders (x^2^_1_ = 13.419, *P* < .001), ages (x^2^_9_ = 34.631, *P* < .001), education levels (x^2^_4_ = 26.656, *P* < .001) and areas of living (x^2^_1_ = 10.557, *P* = .001). With respect to the utilization of PRWs to *select future physicians*, significant effects within the demographic variables gender (x^2^_1_ = 15.451, *P* < .001), age (x^2^_9_ = 18.441, *P* = .030), marital status (x^2^_3_ = 8.207, *P* = .042) and area of living (x^2^_1_ = 7.570, *P* = .006) could be found. Finally, no significant differences regarding the *provision of personal feedback* on PRWS could be demonstrated within the demographic variables. Table 7 compares the results in terms of demographic characteristics associated with the interaction on PRWs.

**Table 7:** Types of interaction with PRWs and association with demographic variables.

*Variables*	*Users n (%)*	*Non Users n (%)*	*Total*	*X ^2^ (df)*	*P*	*V*
***Search for general information***						
***Gender*** ^a^				**4.902 (1)**	**.027**	**.09**
*Female*	264 (86.3)	42 (13.7)	306			
*Male*	198 (79.2)	52 (20.8)	250			
***Age***				**17.483 (9)**	**< .001**	**.18**
*15–19*	54 (72.0)	21 (28.0)	75			
*20–24*	128 (84.2)	24 (15.8)	152			
*25–29*	92 (86.8)	14 (13.2)	106			
*30–34*	69 (88.5)	9 (11.5)	78			
*35–39*	57 (89.1)	7 (10.9)	64			
*40–44*	28 (82.4)	6 (17.6)	34			
*45–49*	12 (75.0)	4 (25.0)	16			
*50–54*	12 (75.0)	4 (25.0)	16			
*55–59*	5 (55.6)	4 (44.4)	9			
*60+*	7 (87.5)	1 (12.5)	8			
***Education***				**1.851 (4)**	**.763**	**.06**
*Compulsory Ed.*	25 (78.1)	7 (21.9)	32			
*Voc. Sec. Ed.*	51 (82.3)	11 (17.7)	62			
*Apprenticeship*	66 (86.8)	10 (13.2)	76			
*High School*	190 (81.9)	42 (18.1)	232			
*Univ. Degree*	132 (84.6)	24 (15.4)	156			
***Marital Status***				**7.024 (3)**	**.071**	**.12**
*Single*	183 (81.0)	43 (19.0)	226			
*Close-partnered*	199 (86.9)	30 (13.1)	229			
*Married*	76 (81.7)	17 (18.3)	93			
*Divorced*	6 (60.0)	4 (40.0)	10			
***Occupation***				**6.213 (4)**	**.184**	**.11**
*Sal. employee*	218 (85.2)	38 (14.8)	256			
*Unemployed*	40 (81.6)	9 (18.4)	49			
*Self-employed*	38 (73.1)	14 (26.9)	52			
*In Training*	160 (82.9)	33 (17.1)	193			
*Retired*	8 (100.0)	0 (0.0)	8			
***Area of Living***				**.90 (1)**	**.764**	**.01**
*Urban*	289 (83.5)	57 (16.5)	346			
*Rural*	175 (82.5)	37 (17.5)	212			
***Variables***	**Users n (%)**	**Non Users n (%)**	**Total**	**X** ^**2**^ **(df)**	**P**	**V**
***Search for physicians***						
***Gender*** ^a^				**6.922 (1)**	**.009**	**.12**
*Female*	247 (80.7)	59 (19.3)	306			
*Male*	178 (71.2)	72 (28.8)	250			
***Age***				**33.638 (9)**	**< .001**	**.25**
*15–19*	40 (53.3)	35 (46.7)	75			
*20–24*	114 (75.0)	38 (25.0)	152			
*25–29*	87 (82.1)	19 (17.9)	106			
*30–34*	68 (85.9)	11 (14.1)	78			
*35–39*	56 (87.5)	8 (12.5)	64			
*40–44*	25 (73.5)	9 (26.5)	34			
*45–49*	13 (81.3)	3 (18.8)	16			
*50–54*	13 (81.3)	3 (18.8)	16			
*55–59*	6 (66.7)	3 (33.3)	9			
*60+*	6 (75.0)	2 (25.0)	8			
***Education***				**6.907 (4)**	**.141**	**.11**
*Compulsory Ed.*	22 (68.8)	10 (31.2)	32			
*Voc. Sec. Ed.*	46 (74.2)	16 (25.8)	62			
*Apprenticeship*	61 (80.3)	15 (19.7)	76			
*High School*	169 (72.8)	63 (27.2)	232			
*Univ. Degree*	129 (82.7)	27 (17.3)	156			
***Marital Status***				**6.898 (3)**	**.075**	**.11**
*Single*	161 (71.2)	65 (28.8)	225			
*Close-partnered*	182 (79.5)	47 (20.5)	229			
*Married*	77 (82.8)	16 (17.2)	93			
*Divorced*	7 (70.0)	3 (30.0)	10			
***Occupation***				**10.723 (4)**	**.030**	**.14**
*Sal. employee*	208 (81.3)	48 (18.8)	256			
*Unemployed*	40 (81.6)	9 (18.4)	49			
*Self-employed*	39 (75.0)	13 (25.0)	52			
*In Training*	133 (68.9)	60 (31.1)	193			
*Retired*	7 (87.5)	1 (12.5)	8			
***Area of Living***				**1.158 (1)**	**.282**	**.05**
*Urban*	270 (78.0)	76 (22.0)				
*Rural*	157 (74.1)	55 (25.9)				
***Variables***	**Users n (%)**	**Non-Users n (%)**	**Total**	**X** ^**2**^ **(df)**	**P**	**V**
***Read physicians’ ratings***						
***Gender*** ^a^				**13.419 (1)**	**< .001**	**.16**
*Female*	207 (61.2)	99 (32.4)	306			
*Male*	131 (52.7)	119 (47.6)	250			
***Age***				**34.631 (9)**	**< .001**	**.25**
*15–19*	31 (41.3)	44 (58.7)	75			
*20–24*	87 (57.2)	65 (42.8)	152			
*25–29*	74 (69.8)	32 (30.2)	106			
*30–34*	59 (75.6)	19 (24.4)	78			
*35–39*	40 (62.5)	24 (37.5)	64			
*40–44*	25 (73.5)	9 (26.5)	34			
*45–49*	11 (68.8)	5 (31.3)	16			
*50–54*	8 (50.0)	8 (50.0)	16			
*55–59*	2 (22.2)	7 (77.8)	9			
*60+*	3 (37.5)	5 (62.5)	8			
***Education***				**26.656 (4)**	**< .001**	**.22**
*Compulsory Ed.*	15 (46.9)	17 (53.1)	32			
*Voc. Sec. Ed.*	37 (59.7)	25 (40.3)	62			
*Apprenticeship*	41 (53.9)	35 (46.1)	76			
*High School*	126 (54.3)	106 (45.7)	232			
*Univ. Degree*	121 (77.6)	35 (22.4)	156			
***Marital Status***				**7.318 (3)**	**.062**	**.12**
*Single*	129 (57.1)	97 (42.9)	225			
*Close-partnered*	149 (65.1)	80 (34.9)	229			
*Married*	59 (36.6)	34 (36.6)	93			
*Divorced*	3 (30.0)	7 (70.0)	10			
***Occupation***				**7.725 (4)**	**.102**	**.12**
*Sal. employee*	167 (65.2)	89 (34.8)	256			
*Unemployed*	34 (69.4)	15 (30.6)	49			
*Self-employed*	30 (57.7)	22 (42.3)	52			
*In Training*	104 (53.9)	89 (46.1)	193			
*Retired*	5 (62.5)	3 (37.5)	8			
***Area of Living***				**10.557 (1)**	**.001**	**.14**
*Urban*	229 (66.2)	117 (33.8)	346			
*Rural*	111 (52.4)	101 (47.6)	212			
***Variables***	**Users n (%)**	**Non-Users n (%)**	**Total**	**X** ^**2**^ **(df)**	**P**	**V**
***Select future physicians***						
***Gender*** ^a^				**15.451 (1)**	**< .001**	**.12**
*Female*	208 (68.0)	98 (32.0)	306			
*Male*	129 (51.6)	121 (48.4)	250			
***Age***				**18.441 (9)**	**.030**	**.18**
*15–19*	32 (42.7)	43 (57.3)	75			
*20–24*	88 (57.9)	64 (42.1)	152			
*25–29*	71 (67.0)	35 (33.0)	106			
*30–34*	51 (65.4)	27 (34.6)	78			
*35–39*	45 (70.3)	19 (29.7)	64			
*40–44*	21 (61.8)	13 (38.2)	34			
*45–49*	10 (62.5)	6 (37.5)	16			
*50–54*	12 (75.0)	4 (25.0)	16			
*55–69*	4 (44.4)	5 (55.6)	9			
*60+*	4 (50)	4 (50)	8			
***Education***				**8.548 (4)**	**.073**	**.12**
*Compulsory Ed.*	15 (46.9)	17 (53.1)	32			
*Voc. Sec. Ed.*	34 (54.8)	28 (45.2)	62			
*Apprenticeship*	48 (63.2)	28 (36.8)	76			
*High School*	134 (57.8)	98 (42.2)	232			
*Univ. Degree*	107 (68.6)	49 (31.4)	156			
***Marital Status***				**8.207 (3)**	**.042**	**.12**
*Single*	124 (54.9)	102 (45.1)	225			
*Close-partnered*	152 (66.4)	77 (33.6)	229			
*Married*	58 (62.4)	35 (37.6)	93			
*Divorced*	4 (40.0)	6 (60.0)	10			
***Occupation***				**1.614 (4)**	**.806**	**.05**
*Sal. employee*	160 (53.5)	96 (37.5)	256			
*Unemployed*	31 (63.3)	18 (36.7)	49			
*Self-employed*	32 (61.5)	20 (38.5)	52			
*In Training*	110 (57.0)	83 (43.0)	193			
*Retired*	5 (62.5)	3 (37.5)	8			
***Area of Living***				**7.570 (1)**	**.006**	**.116**
*Urban*	225 (65.0)	121 (35.0)	346			
*Rural*	113 (53.3)	99 (46.7)	212			
***Variables***	**Aware n (%)**	**Not Aware n (%)**	**Total**	**X** ^**2**^ **(df)**	**P**	**V**
***Provide personal feedback***						
***Gender*** ^a^				**.001 (1)**	**.975**	**.00**
*Female*	45 (14.7)	261 (85.3)	306			
*Male*	37 (14.8)	213 (85.2)	250			
***Age***				**13.797 (9)**	**.130**	**.157**
*15–19*	7 (9.3)	68 (90.7)	75			
*20–24*	19 (12.5)	133 (87.5)	152			
*25–29*	23 (21.7)	83 (78.3)	106			
*30–34*	9 (11.5)	69 (88.5)	78			
*35–39*	15 (23.4)	49 (76.6)	64			
*40–44*	4 (11.8)	30 (88.2)	34			
*45–49*	1 (6.3)	15 (93.8)	16			
*50–54*	3 (18.8)	13 (81.3)	16			
*55–59*	1 (11.1)	8 (88.9)	9			
*60+*	0 (0.0)	8 (100.0)	8			
***Education***				**2.314 (4)**	**.678**	**.06**
*Compulsory Ed.*	3 (9.4)	29 (90.6)	32			
*Voc. Sec. Ed.*	11 (17.7)	51 (82.3)	62			
*Apprenticeship*	12 (15.8)	64 (84.2)	76			
*High School*	30 (12.9)	202 (87.1)	232			
*Univ. Degree*	26 (16.7)	130 (83.3)	156			
***Marital Status***				**.268 (3)**	**.966**	**.02**
*Single*	33 (14.6)	193 (85.4)	225			
*Close-partnered*	34 (14.8)	195 (85.2)	229			
*Married*	13 (14.5)	80 (86.0)	93			
*Divorced*	2 (20.0)	8 (80.0)	10			
***Occupation***				**2.010 (4)**	**.734**	**.06**
*Sal. employee*	39 (15.2)	217 (84.8)				
*Unemployed*	10 (20.4)	39 (79.6)				
*Self-employed*	6 (11.5)	46 (88.5)				
*In Training*	26 (13.5)	167 (86.5)				
*Retired*	1 (12.5)	7 (87.5)				
***Area of Living***				**3.106 (1)**	**.078**	**.08**
*Urban*	58 (16.8)	288 (83.2)	346			
*Rural*	24 (11.3)	188 (88.7)	212			

^a^ = without diverse gender (n = 2)

With respect to psychographic characteristics and interaction with PRWs, significant mean differences were found for four of the five interaction types. Respondents who used PRWs to *search for general information* were characterized by better *feelings towards the internet* (t_556_ = 2.30, *P* = .022), a higher *digital literacy* (t_556_ = 2.64, *P* = .008) as well as a higher *eHealth literacy* (t_116_ = 4.56, *P* < .001). In addition to that, participants who used PRWs to *search for physicians* were characterized by a higher *eHealth literacy* (t_187_ = 3.24, *P* = .001). Respondents who used PRWs to *read physicians’ ratings* were also characterized by a higher *eHealth literacy* (t_391_ = 4.034, *P* < .001). Finally, participants who used PRWs to *select future physicians* were characterized by better *feelings towards the internet* (t_556_ = 2.549, *P* = .011) as well as a higher *eHealth literacy* (t_410_ = 3.650, *P* < .001). No significant differences were detected when focusing on psychographic characteristics and the utilization of PRWs to *provide personal feedback*. Additionally, *review skepticism* had no significant impact on the different types of interaction, even though non-users revealed a higher level of review skepticism than users by trend. An overview regarding psychographic characteristics related to the interaction with PRWs can be found in Table 8.

**Table 8:** Types of interaction with PRWs and association with psychographic variables.

***Search for general information***						
***Variables***	**Users (n = 464)**		**Nonusers (n = 94)**		**T (df)**	**P**
	**Mean**	**SD**	**Mean**	**SD**		
Feelings towards the internet*	5.49	1.13	5.19	1.19	2.303 (556)	.022
Digital literacy**	6.22	.92	5.94	1.10	2.641 (556)	.008
eHealth literacy**	5.54	.94	4.93	1.22	4.560 (116)	< .001
Review skepticism***	4.07	1.29	4.23	1.33	-1.114 (556)	.266
***Search for physicians***						
***Variables***	**Users (n = 427)**		**Nonusers (n = 131)**		**T (df)**	**P**
	**Mean**	**SD**	**Mean**	**SD**		
Feelings towards the internet*	5.45	1.14	5.39	1.16	.567 (556)	.571
Digital literacy**	6.19	.95	6.13	.97	.579 (556)	.563
eHealth literacy**	5.52	.96	5.16	1.15	3.241 (187)	.001
Review skepticism***	4.08	1.27	4.16	1.38	-.627 (556)	.531
***Read physicians’ ratings***						
***Variables***	**Users (n = 340)**		**Nonusers (n = 218)**		**T (df)**	**P**
	**Mean**	**SD**	**Mean**	**SD**		
Feelings towards the internet*	5.48	1.15	5.38	1.14	1.037 (556)	.300
Digital literacy**	6.24	.92	6.07	1.00	1.960 (556)	.051
eHealth literacy**	5.58	.91	5.21	1.13	4.034 (391)	< .001
Review skepticism***	4.02	1.30	4.21	1.29	-1.766 (556)	.078
***Select future physicians***						
***Variables***	**Users (n = 338)**		**Nonusers (n = 220)**		**T (df)**	**P**
	**Mean**	**SD**	**Mean**	**SD**		
Feelings towards the internet*	5.54	1.127	5.29	1.164	2.549 (556)	.011
Digital literacy**	6.20	.911	6.13	1.018	.803 (556)	.422
eHealth literacy**	5.56	.931	5.23	1.11	3.650 (410)	< .001
Review skepticism***	4.03	1.27	4.20	1.33	-1.531 (556)	.126
***Provide personal feedback***						
***Variables***	**Users (n = 82)**		**Nonusers (n = 476)**		**T (df)**	**P**
	**Mean**	**SD**	**Mean**	**SD**		
Feelings towards the internet*	5.37	1.18	5.45	1.142	-.625 (556)	.532
Digital literacy**	6.20	.99	6.17	.95	.237 (556)	.813
eHealth literacy**	5.57	.98	5.41	1.02	1.269 (556)	.205
Review skepticism***	3.86	1.34	1.29	4.14	-1.758 (556)	.079

* The scale options range from 1 (“very negative feelings”) to 7 (“very positive feelings”).

** The scale options range from 1 (“not literate at all”) to 7 (“very literate”).

*** The scale options range from 1 (“not sceptic at all”) to 7 (“very sceptic”).

## Discussion

### Principal results

In general, three core contributions could be achieved by using the results of the current study. First of all, it is the first study ever to deal with PRWs in Austria. However, the results should also be of considerable interest for the observation and further development of international PRW as well as rating and review website research. In this context, we were able to investigate the influence of demographic and psychographic factors on awareness of, and interaction with PRWs. Furthermore, we were able to show that there is not one type of interaction on PRWs, but several different forms of interaction. In this context, these forms of interaction are not only characterized by different usage rates, but also by individual factors influencing the respective use behavior.

### General awareness (RQ1)

Within our study sample, *general awareness* regarding PRWs was significantly above the awareness level of the studies by Patel et al. (2018) [54] (15.2%), Terlutter et al. (2014) [58] (29.3%), and Emmert et al. (2013) [51] (32.09%). Similar levels were measured in the studies of Hanauer et al. (2014) [50] (65.0%), McLennan et al. (2019) [33] (72.5%) and Hanauer et al. (2014) [78] (74.0%).

Even though the *general awareness* of PRWs seems to be relatively high within the study population (75.6%), there are significant differences regarding the awareness level within various demographic and psychographic characteristics. Women generally had a higher awareness level than men. A similar relationship was demonstrated within the studies by McLennan et al. (2017) [33] and Emmert et al. (2013) [51]. In addition to that, we were able to show that there is a significant difference regarding *general awareness* of PRWs between participants in different areas of living. In this context, respondents living in more urban areas had a higher awareness level than respondents living in more rural areas. One reason for this effect could be that urban areas are characterized by a higher level of anonymity [90] and therefore PRWs are more likely to be consulted as health specific advisors than family and friends. Additionally, respondents with a higher digital as well as a higher eHealth literacy were also characterized by a higher awareness level regarding PRWs. A comparable result was obtained by Emmert et al. (2013) [51]. This situation may be related to the fact that these groups of people are characterized by a more pronounced online behavior, which also leads to a stronger expression of PRWs *general awareness*.

### Awareness compared to alternative rating portals (RQ2)

In RQ2 we tried to discover whether the level of awareness is high or low compared to alternative rating websites. Although PRWs are known by more than three quarters of the study population, they seem to remain in the lower midfield in terms of *awareness compared to other rating portals*. Similarly, in the McLennan et al. (2017) [33] study, awareness of PRWs was estimated to be relatively low compared to alternative review portals. The low *awareness compared to alternative rating portals* could be due to the fact that some of those alternative websites have been available for a very long time and thus the market penetration seems to be more pronounced (see e.g., [108–110]). Conversely, this circumstance could also be a reason for the lower awareness of rather novel online portals like real estate rating websites (61.1%), teacher rating websites (48.7%) or hospital rating websites (44.6%).

### Brand awareness of specific PRWs (RQ3)

In terms of unaided *brand awareness* (*recall*), Docfinder.at received the highest level of brand awareness within the study population. The subsequent *brand recall* areas are occupied by the evaluation areas of Google.at and Herold.at. A similar picture emerges in the *brand awareness* study part. Even in this area, Docfinder.at, Google.at and Herold.at occupy ranks 1–3. In particular, the high recall value of 31.36% shows that Docfinder.com appears to be the best-known brand in the Austrian PRWs sector. This fact is reinforced by the relatively high gap of 23.12 percentage points to second place in the recall area and 7.5 percentage points to second place in the recognition area.

### Interaction with physician rating websites (RQ4)

Interaction with PRWs seems to be uniformly high (89.2%). The interaction level is thus significantly higher than in the studies already published, which showed levels between 0.4% in England (2018) [54] and 39% in the United States of America (2017) [52]. We attribute the fact that the interaction level is higher than the awareness level (75.6%) to respondents having used PRWs in the past without being aware of it. This conclusion is also supported by the collected data. The highest interaction levels were found for using those websites to *search for general information* (83.2%) and to *search for physicians* (76.5%). These types of interaction are not PRW specific and can also be conducted via health-specific websites or search engines. After the *search for general information* and the *search for physicians*, a PRW-specific form of interaction occurs with the *reading of physicians’ ratings*. More than half of the study participants reported using PRWs for this type of interaction. Since this is a very specific form of interaction, it can be assumed that people who use PRWs to *read physicians’ ratings* are aware of the platform itself, while the *search for general information* and the *search for physicians* can also be carried out unconsciously on PRWs. While 60.9% of the study participants indicated that they use PRWs to read physicians’ ratings and 60.6% used these portals to *select future physicians*, the number of respondents who also *provided personal feedback* online was relatively low (14.7%). This result is in line with the body of literature that has shown that while people frequently read online reviews, they rarely post reviews (see e.g., [111–114]).

Besides the results regarding the general interaction with PRWs, differences in the interaction types with PRWs between several demographic groups could be demonstrated. In this context, women were more likely to use PRWs to *search for general information*, to *search for physicians*, to *read physicians’ ratings* and to *select future physicians* than men. Similar results were presented by Emmert et al. (2013) [51] and Terlutter et al. (2014) [58] who also found a higher general interaction with PRWs by female respondents. In addition to that, different age groups are also characterized by a varying use behavior. Thus, it was shown that in the youngest age group fewer people used PRWs to *search for general information*, to *search for physicians*, to *read physicians’ ratings* and to *select future physicians* than in the older age groups. This circumstance could be due to the fact that younger persons and young adults are often characterized by a rather risky health behavior and accordingly do not deal sufficiently with health-specific issues (see e.g., [115–117]). Furthermore, differences in the area of living were also found. Thus, it was shown that more people living in urban regions use PRWs to *read physicians’ ratings* and *select future physicians* than people living in rural regions. This result supports our assumption that PRWs are used more heavily in urban regions than in rural regions. One reason for this could be the higher level of anonymity in urban regions [90]. Since the relationship with family and friends is not as established in cities as in rural areas, alternative information channels such as PRWs are more likely to be used as health specific information source.

In addition to that, occupation had an impact on whether respondents used PRWs to *search for physicians*. In that respect, it was observed that more salaried employees, unemployed participants, retired respondents and self-employed study participants use PRWs to *search for physicians* than study participants who are currently in training, such as pupils or students. Since it can be assumed that a large proportion of the study participants who are currently in training belong to the younger age group, we again attribute the lower use to the rather risky health behavior of this age group (see e.g., [115–117]).

When focusing on the psychographic variables, it was found that *eHealth literacy* seems to have an influence on the likelihood of PRWs use. In contrast to that, a relation between digital literacy and the use of PRWs was only found for one category of use. Respondents with higher digital literacy were more likely to use PRWs to *search for general information* than respondents with lower digital literacy. A similar result was demonstrated in the study by Hanauer et al. (2014) [78]. Finally, it was shown that respondents with better *feelings towards the internet* had a higher probability to use PRWs to *search for general information* and *select future physicians* than respondents with worse *feelings towards the internet*.

### Limitations

Some limitations could be identified within this study. As mentioned in the methods section, especially within online surveys it must always be expected that study participants are not very attentive, show acquiescence bias (propensity to agree or disagree with almost all questionnaire items) or extremity bias (propensity to select bend points) [118]. As described, we tried to limit these risks by using logic tasks as well as attention checks and by removing completed questionnaires including a conspicuous response behavior. In addition to that, participants of an online survey may have a more comprehensive understanding of online topics. This may have led to a more pronounced representation of awareness of and interaction with PRWs within the study population. Another limitation can be found in the scope of the survey instrument. Although we have expanded and adapted the scales used, they still do not cover all potential areas of investigation. For example, when comparing different rating portals, additional categories can also be included. As we used a quantitative research setting, we did not focus on identifying reasons for the use or non-use of PRWs. Future studies could therefore also focus on the barriers and facilitators of PRW usage. By using Clickworker.com as an online tool for conducting the study, it was possible to reach a large number of individuals. Nevertheless, it must be pointed out at this point that this excluded people without Clickworker.com accounts from participating. Finally, our study results may not represent the entire Austrian population, as our study participants were predominantly more highly educated, female and middle-aged.

### Practical implications

Considering the high level of awareness of and interaction with PRWs within the study population, it can be assumed that these platforms have become the first port of call when searching for physicians in the digital environment. This circumstance shows that these portals represent the physician as well as the medical practice to the outside world and for this reason PRWs should be maintained and serviced accordingly by physicians. In contrast to the external effect, PRWs also have a more internal aspect. 14.7% of our study population stated that they had already rated a physician. This result indicates that certain aspects, which otherwise would have to be investigated by market research processes, can be represented on PRWs. These include, for example, the survey of patient satisfaction, the search for critical incidents or the search for indications for the improvement of process-related performance in the medical practice. This demonstrates that PRWs can be used as a cost-effective market research tool from a physician’s perspective. It has to be noted, however, that a sufficient number of reviews should be in place to ensure an objective representation of the medical service performance.

Results show that care should be taken when using ratings to build expectations regarding a future visit of a specific physician. Although many patients read ratings, they are only written by a relatively small proportion of the general population. For this reason, it is necessary to ensure that there are enough reviews to draw a representative digital picture of the physician’s practice.

The present study shows that PRWs are already known by the majority of the study population and that these portals are used by more than half of them. This can basically be interpreted as a good sign for maintainers of PRWs. However, the rating function should be used by significantly more patients to increase the use and trustworthiness of PRWs by both, physicians and patients. It is therefore advisable to develop strategies to increase the willingness of patients to rate physicians on PRWs.

Even for policy makers the results of the current study could support their future decision-making behavior. As we have shown, different levels of digital and eHealth literacy are present in the population. The promotion of further education in the field of digital and eHealth literacy could therefore contribute to people feeling more confident when using digital media and being able to distinguish true information from false information.

## Conclusions

Our results have led to a number of relevant findings that grant deeper insights into whether and how different population segments have used PRWs so far. According to the sample used in the current study, the majority of the study population is aware that PRWs exist. Significant correlations regarding the *general awareness* of PRWs were found within the demographic variables gender, age, education, occupation and area of living as well as for respondents reporting a higher digital and eHealth literacy. Notwithstanding, compared to the *awareness regarding alternative rating websites*, PRWs are still in the lower midfield, as the upper ranks are occupied by restaurant rating websites, hotel rating websites or movie rating websites. In terms of *recall* and *recognition*, the Austrian PRW Docfinder.at received the highest level of *brand awareness*, followed by the rating websites of Google.at and Herold.at. Besides the results with respect to awareness, the usage of PRWs is also characterized by a high level of interaction. Significant correlations were found within most of the investigated demographic and psychographic variables. Even though our results suggest a high *awareness of and interaction with PRWs* across the entire population, it seems to be most pronounced among female, middle-aged, more highly educated and urban population segments. Moreover, feelings towards the internet, digital- as well as- eHealth literacy seem to play an important role in the PRWs use behavior. This shows that PRWs represent an effective tool for sustained interaction with female, middle-aged, higher educated, urban and digitally literate population segments. Given the high level of awareness and interaction regarding PRWs within our study population, however, PRWs seem to represent an effective tool for health related communication, at least in the long run.

## Supporting information

S1 File Questionnaire PRWs. (PDF)

S2 File Example equation, introduction text and attention check. (PDF)

S3 File Data selection process. (PDF)

## Abbreviations

Compulsory Ed.: Compulsory Education
eHealth: electronic Health
MTurk: Mechanical Turk
PRW: Physician Rating Website
PRWs: Physician Rating Websites
Sal. employee: Salaried employee
Voc. Sec. Ed.: Vocational Secondary Education
