# Supplementary material for: Awareness of and interaction with physician rating websites: A cross-sectional study in Austria
Source: PLoS One. 2022 Dec 30;17(12):e0278510. doi: 10.1371/journal.pone.0278510 (PMC9803240; doi:10.1371/journal.pone.0278510)
Supplement: S1 File — (PDF) [file pone.0278510.s001.pdf]

Variable	Sources	Original measurement	Measurement adapted for research study
**1. Demographic & Psychographic Variables**			
**Gender**<br><br>**(Gen)**	[15]	Gender<br><br>1. Male<br>2. Female	Gender<br><br>1. Male<br>2. Female<br>3. Diverse
**Age**<br><br>**(Age)**	[15]	Year of Birth:	Year of Birth:
**Education**<br><br>**(Edu)**	[15]	Highest level of education:<br>1. Without school qualification<br>2. Secondary general school<br>3. Polytechnic secondary school<br>4. Intermediate secondary school<br>5. High school diploma / A-levels<br>6. University degree<br>7. Postdoctoral degree / Professor	Highest level of education:<br>1. Without school qualification<br>2. Secondary general school<br>3. Polytechnic secondary school<br>4. Intermediate secondary school<br>5. High school diploma / A-levels<br>6. University degree<br>7. Postdoctoral degree / Professor
**Marital Status**<br><br>**(Mas)**	[15]	Marital status:<br>1. Single<br>2. Close-partnered<br>3. Married<br>4. Divorced<br>5. Widowed<br>6. No answer	Marital status:<br>1. Single<br>2. Close-partnered<br>3. Married<br>4. Divorced<br>5. Widowed<br>6. Other

**Occupation**<br><br>**(Occ)**	[51]	Please indicate your current occupation:<br>1. Self-employed<br>2. Civil servant<br>3. Employee<br>4. Apprentice<br>5. Unemployed<br>6. Pensioner<br>7. Student (pupil)<br>8. Student (Univ./tech. univ.)<br>9. Other<br>10. I do not wish to answer this question	Please indicate your current occupation:<br>1. Self-employed<br>2. Civil servant<br>3. Employee<br>4. Apprentice<br>5. Unemployed<br>6. Pensioner<br>7. Student (pupil)<br>8. Student (Univ./tech. univ.)<br>9. Other
**Area of Living**<br><br>**(AoL)**	[100]	Area of Residence:<br>1. Urban<br>2. Rural	Area of Residence:<br>1. Urban<br>2. Rural
**Feelings twd. t. Int.**<br><br>**(FtI)**	[58]	What kind of feelings do you have toward the internet and other web---based applications (e.g. apps on the smart phone or tablet) in general?	What kind of feelings do you have toward the internet and other web---based applications (e.g. apps on the smart phone or tablet) in general?
**Digital Literacy**<br><br>**(Dlit)**	[58]	How would you rate your own internet skills on a scale from 1 (I am not literate at all) to 7 (I am very literate)?	How would you rate your own internet skills on a scale from 1 (I am not literate at all) to 7 (I am very literate)?

**E- Health Literacy**<br><br>**(EHL)**	[101]<br><br>[102]	The scale options ranged from 1 (“strongly disagree”) to 5 (“strongly agree”).<br><br>1. I know how to find helpful health resources on the internet<br>2. I know how to use the internet to answer my health questions<br>3. I know what health resources are available on the internet<br>4. I know where to find helpful health resources on the internet<br>5. I know how to use the health information I find on the internet to help me<br>6. I have the skills I need to evaluate the health resources I find on the internet<br>7. I can tell high quality from low quality health resources on the internet	The scale options range from 1 (“strongly disagree”) to 7 (“strongly agree”).<br><br>1. I know how to find helpful health resources on the internet<br>2. I know how to use the internet to answer my health questions<br>3. I know what health resources are available on the internet<br>4. I know where to find helpful health resources on the internet<br>5. I know how to use the health information I find on the internet to help me<br>6. I have the skills I need to evaluate the health resources I find on the internet<br>7. I can tell high quality from low quality health resources on the internet
**O.R. Scepticism**<br><br>**(RevScep)**	[103]	The scale options ranged from 1 (“strongly disagree”) to 5 (“strongly agree”).<br>• I am basically doubtful about online reviews.<br>• Online reviews are often questionable.<br>• I am generally uncertain about online reviews.<br>• I am generally sceptical about online reviews.	The scale options range from 1 (“strongly disagree”) to 7 (“strongly agree”).<br>• I am basically doubtful about online reviews.<br>• Online reviews are often questionable.<br>• I am generally uncertain about online reviews.<br>• I am generally sceptical about online reviews.
**2. Online Rating & Review Websites**			
**Awareness**<br><br>**(Awa)**	[50]	Multiple Response YN Scale<br><br>1. Are you aware that there are websites that rate and review the following?<br>Cars – Movies or Books – Electronics or Appliances – Restaurants – Other – Physicians – Hospitals - Dentists	Multiple Response YN Scale<br><br>1. Are you aware that there are websites that rate and review the following?<br>Leisure Activities - Cars – Movies - Books – Electronics – Restaurants – Physicians – Hospitals - Teachers – Employers – Clothing – Accommodation Services – Real Estate - Other
**Recall**<br><br>**(BraReca)**	[104]	Open Question Format<br>What brands of the product or service category can you think of?	Open Question Format<br>What PRWs can you think of?

<table>
<tr><th colspan="4">3. Physician Rating Websites</th></tr>
<tr><td>Attention Check

(ACH)</td><td>[105]
[106]</td><td colspan="2">Physician Rating Websites offer healthcare consumers the opportunity to anonymously rate their doctor. These ratings could help future or potential patients in the decision making process regarding their future medical care. To continue our research focusing on physician rating portals, we would like to learn more about you and your interaction with those online platforms. As a first step, we are interested in whether you take the time to read the text content comprehensively. To confirm that you have read the instructions, please ignore the next question and select only the option 'Other'.
Which state do you come from?
o Burgenland
o Carinthia
o Lower Austria
o Upper Austria
o Salzburg
o Styria
o Tyrol
o Vorarlberg
o Vienna
o Other</td></tr>
<tr><td>Recognition

(BraReco)</td><td>[51]</td><td>Multiple Response Scale 1. Have you ever heard of any of the following PRWs?

Sanego.de - Docinsider.de - Jameda.de - Esando.de - Onmeda.de - Imedo.de - Medführer.de - Weisse Liste (Arztnavigator) - Arzt-auskunft.de - I don´t know any of these websites.</td><td>Multiple Response Scale 1. Have you ever heard of any of the following PRWs?

DocFinder.at – Arztsuche24.at – Doc-suche.at – Guetgemacht.at – Google.at (as PRWs) – Herold.at (as PRWs) – FirmenABC (as PRWs) – Praxisplan.at – Tupalo.at (as PRWs) – Susi.at (as PRWs)… - Oher</td></tr>
</table>

**Interaction**<br><br>**(Int)**	[20]	Multiple Response Scale<br>Please indicate your type of activity involving the physician internet rating site.<br>• Reading patient/family reviews of physician(s) only<br>• Providing personal feedback on physician(s) only<br>• Both<br>• Prefer not to answer	Multiple Response Scale<br>Please indicate your type of activity involving physician rating websites.<br>• Reading patient reviews of physician(s)<br>• Providing personal feedback on physician(s)<br>• Search for physician(s)<br>• Select physician(s)<br>• Search for additional information (e.g. telephone number, address…)
