# Supplementary material for: Awareness of and interaction with physician rating websites: A cross-sectional study in Austria
Source: PLoS One. 2022 Dec 30;17(12):e0278510. doi: 10.1371/journal.pone.0278510 (PMC9803240; doi:10.1371/journal.pone.0278510)
Supplement: S2 File — (PDF) [file pone.0278510.s002.pdf]

Equation Example:

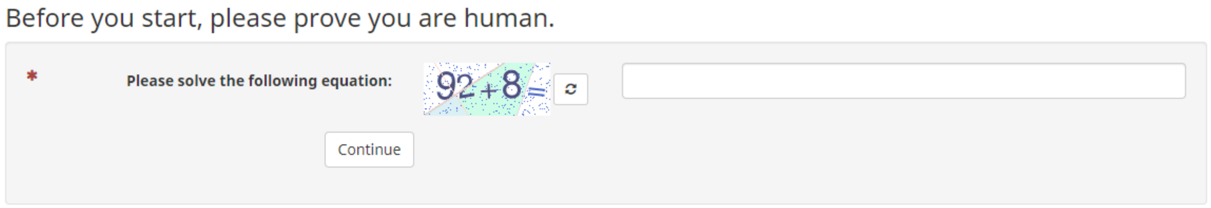

Attention Check:

Physician Rating Websites offer healthcare consumers the opportunity to anonymously rate their doctor. These ratings could help future or potential patients in the decision making process regarding their future medical care. To continue our research focusing on physician rating portals, we would like to learn more about you and your interaction with those online platforms. As a first step, we are interested in whether you take the time to read the text content comprehensively. To confirm that you have read the instructions, please ignore the next question and select only the option 'Other'.

<u>Which state do you come from?</u>

- ○ **Burgenland**
- ○ **Carinthia**
- ○ **Lower Austria**
- ○ **Upper Austria**
- ○ **Salzburg**
- ○ **Styria**
- ○ **Tyrol**
- ○ **Vorarlberg**
- ○ **Vienna**
- ○ **Other**
