# Supplementary figures and images for: Awareness of and interaction with physician rating websites: A cross-sectional study in Austria
Source: PLoS One. 2022 Dec 30;17(12):e0278510. doi: 10.1371/journal.pone.0278510 (PMC9803240; doi:10.1371/journal.pone.0278510)

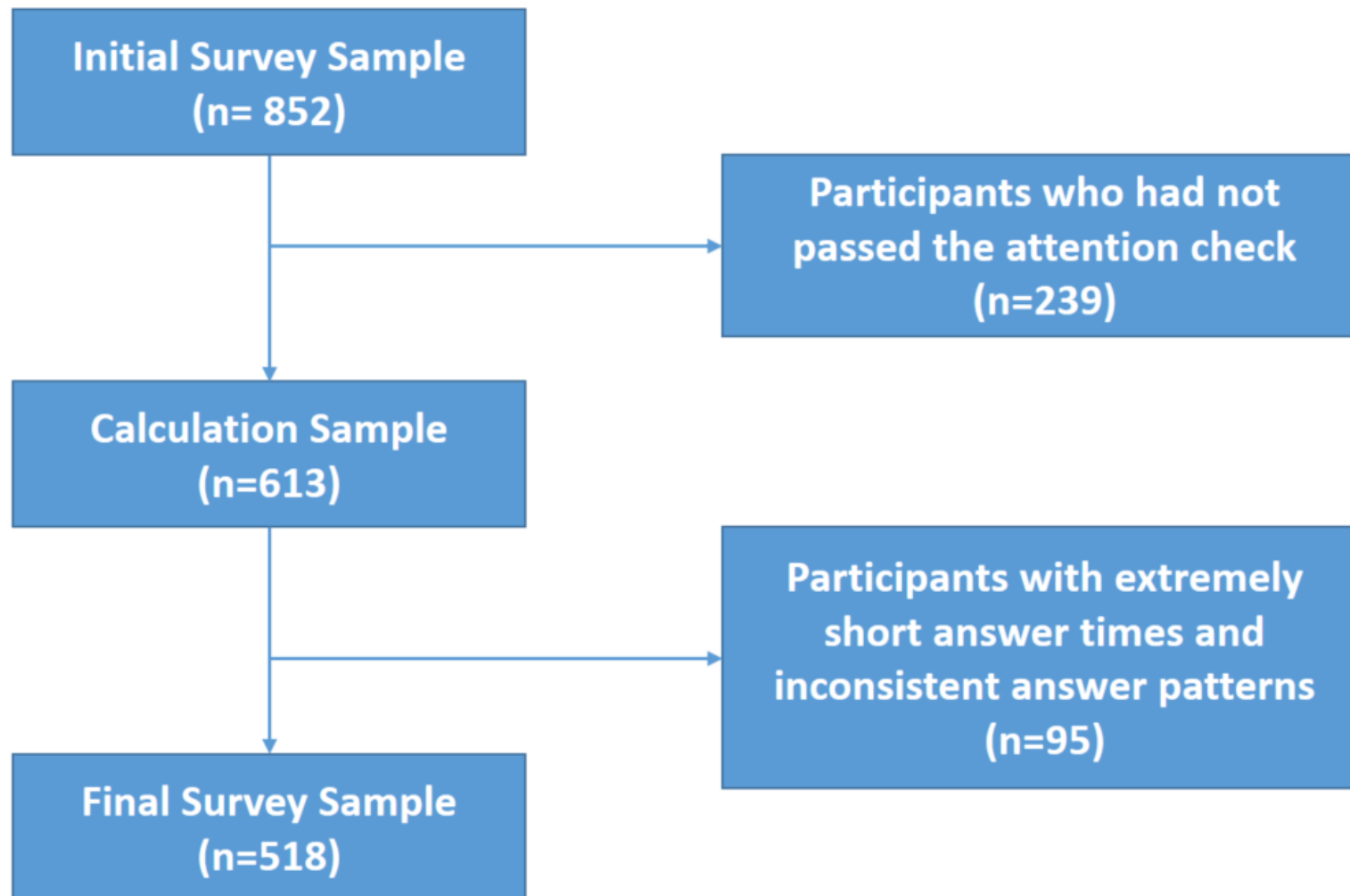

Supplement: S3 File — (PDF) [file pone.0278510.s003.pdf]
